# Case report: Cerebral autosomal dominant arteriopathy with subcortical infarcts and leucoencephalopathy (CADASIL) as a risk factor for central serous chorioretinopathy

**DOI:** 10.3389/fneur.2022.1034718

**Published:** 2022-11-21

**Authors:** Alberto Pazzaglia, Nicola Valsecchi, Matteo Belletti, Fabio Guaraldi, Michela Fresina, Luigi Fontana

**Affiliations:** IRCSS Azienda Ospedaliero-Universitaria di Bologna, Bologna, Italy

**Keywords:** case report, CADASIL, central serous chorioretinopathy, choriocapillaris ischemia, transfoveal subthreshold micropulse yellow laser, atypical case

## Abstract

**Purpose:**

To describe an atypical case of central serous chorioretinopathy (CSC) in a patient with cerebral autosomal dominant arteriopathy with subcortical infarcts and leucoencephalopathy (CADASIL).

**Methods:**

A retrospective case report.

**Results:**

A 43-year-old white man with a genetic diagnosis of CADASIL was referred to our hospital because of reduced visual acuity in his right eye (20/30). In the previous 2 months, he developed CSC with subretinal fluid (SRF) and damage to the retinal pigmented epithelium without pachychoroid and pachyvessels or known risk factors for CSC. The patient was treated with transfoveal subthreshold micropulse yellow laser (577 nm) therapy. One month later, there were no signs of SRF, and visual acuity improved to 20/20.

**Conclusions:**

Cerebral autosomal dominant arteriopathy with subcortical infarcts and leucoencephalopathy **(**CADASIL) is a genetic condition that primarily affects vascular smooth cells in small cerebral vessels and retinal arterioles. However, we hypothesize that CADASIL could also be responsible for an alteration of the vascular smooth cells in the choroidal arterioles, leading to choriocapillaris ischemia and CSC, even in the absence of a pachychoroid spectrum.

## Introduction

Cerebral autosomal dominant arteriopathy with subcortical infarcts and leucoencephalopathy (CADASIL) is a form of small cerebral vessel disease pathology characterized by white matter lesions in the brain and an increased risk for ischemic stroke.

Patients with CADASIL present a mutation in the Notch3 gene on chromosome 19 that encodes a cell surface receptor expressed in vascular smooth cells and pericytes (1). A mutated version of the gene determines an accumulation of the extracellular domain of Notch3 in vascular smooth cells, determining fibrosis and lumen occlusion.

Herein, we describe the case of a patient with a genetic diagnosis of CADASIL that develops central serous chorioretinopathy (CSC) in his right eye in the absence of other risk factors.

To the best of our knowledge, this is the first case reported in the literature of CSC in a patient with CADASIL.

## Case report

A 43-year-old Italian white man was referred to the ophthalmic emergency room at Sant'Orsola-Malpighi Hospital in Bologna with a history of 2 months of blurred vision in his right eye.

He had a genetically confirmed diagnosis of CADASIL, with mutation C.3016C>T in the exon 19 of gene Notch3, responsible for the protein change p.Arg1006Cys, for which he was regularly taking antiplatelet therapy. He had no neurological signs or symptoms, and he reported no previous episodes of aura with or without migraine, diplopia, oscillopsia, or episodes of transient vision loss.

**Figure 1:**
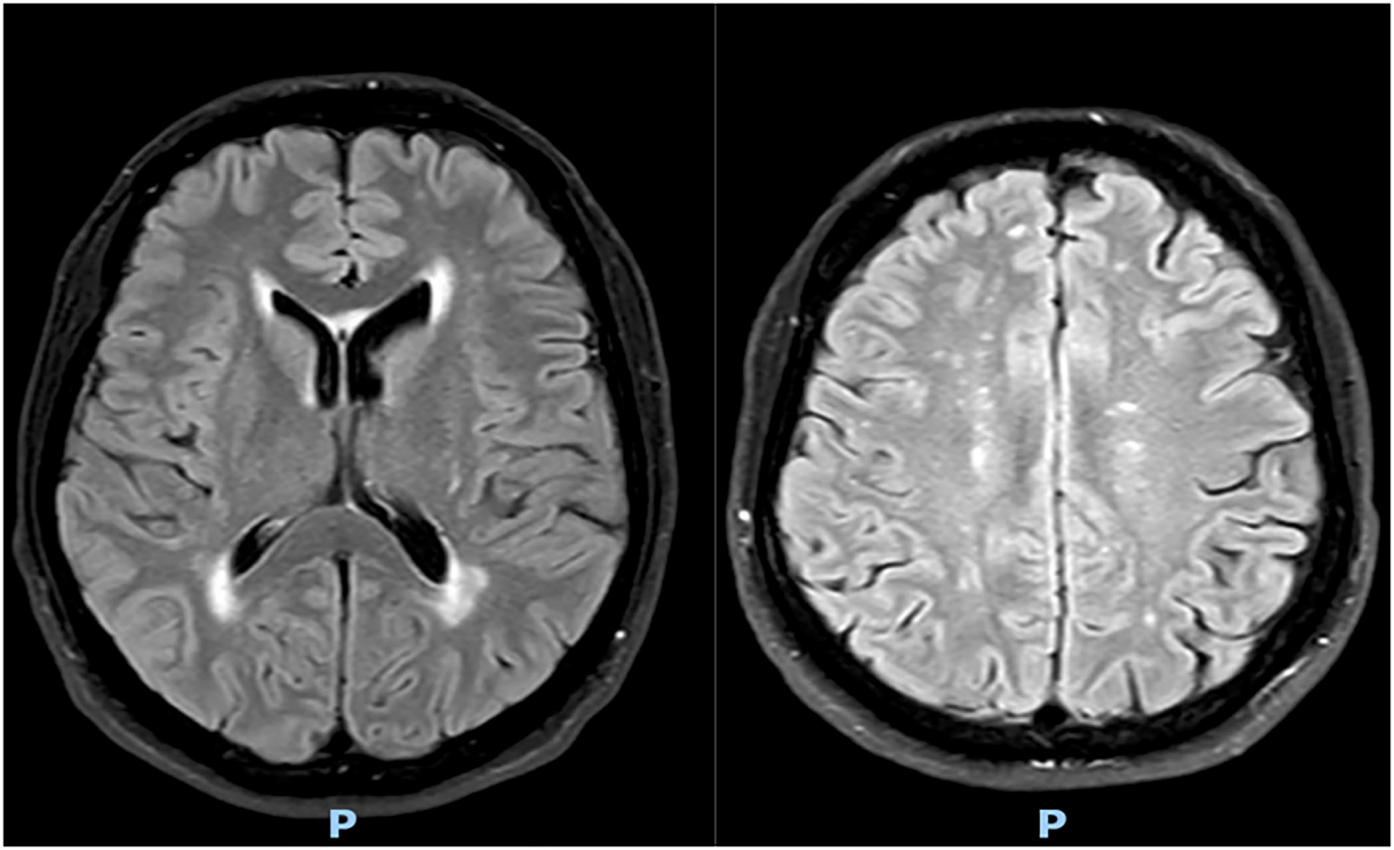
Brain MRI showed multiple bilateral subcortical white matter lesions.

Brain magnetic resonance imaging (MRI) performed 1 month ago showed multiple bilateral subcortical white matter lesions, with no signs of recent ischemia (see Figure 1).

He had no known risk factors for CSC, and he reported no previous history of glucocorticoid intake or stressing conditions. At the ophthalmic evaluation, he presented with a visual acuity of 20/25 in his right eye with low myopia (two diopters), without any other symptoms or complaints. He had no pain at ocular movements, and no relative afferent pupillary defects were present. A careful examination of the anterior and posterior segments was conducted without any clinically meaningful findings. Visual evoked potentials (VEP), electroretinogram (ERG), and computerized visual field did not show any signs of disease.

Spectral domain optical coherence tomography (SD-OCT) (Spectralis OCT, Heidelberg Engineering, Germany) was performed, and an initial subfoveal serous detachment of the neurosensory retina with alteration of the retinal pigmented epithelium (RPE) was found in his right eye, with no signs of pachychoroid and pachyvessels. The left eye macula and the optic nerves of both eyes were not involved.

No treatment was indicated, and a follow-up visit was scheduled.

**Figure 2:**
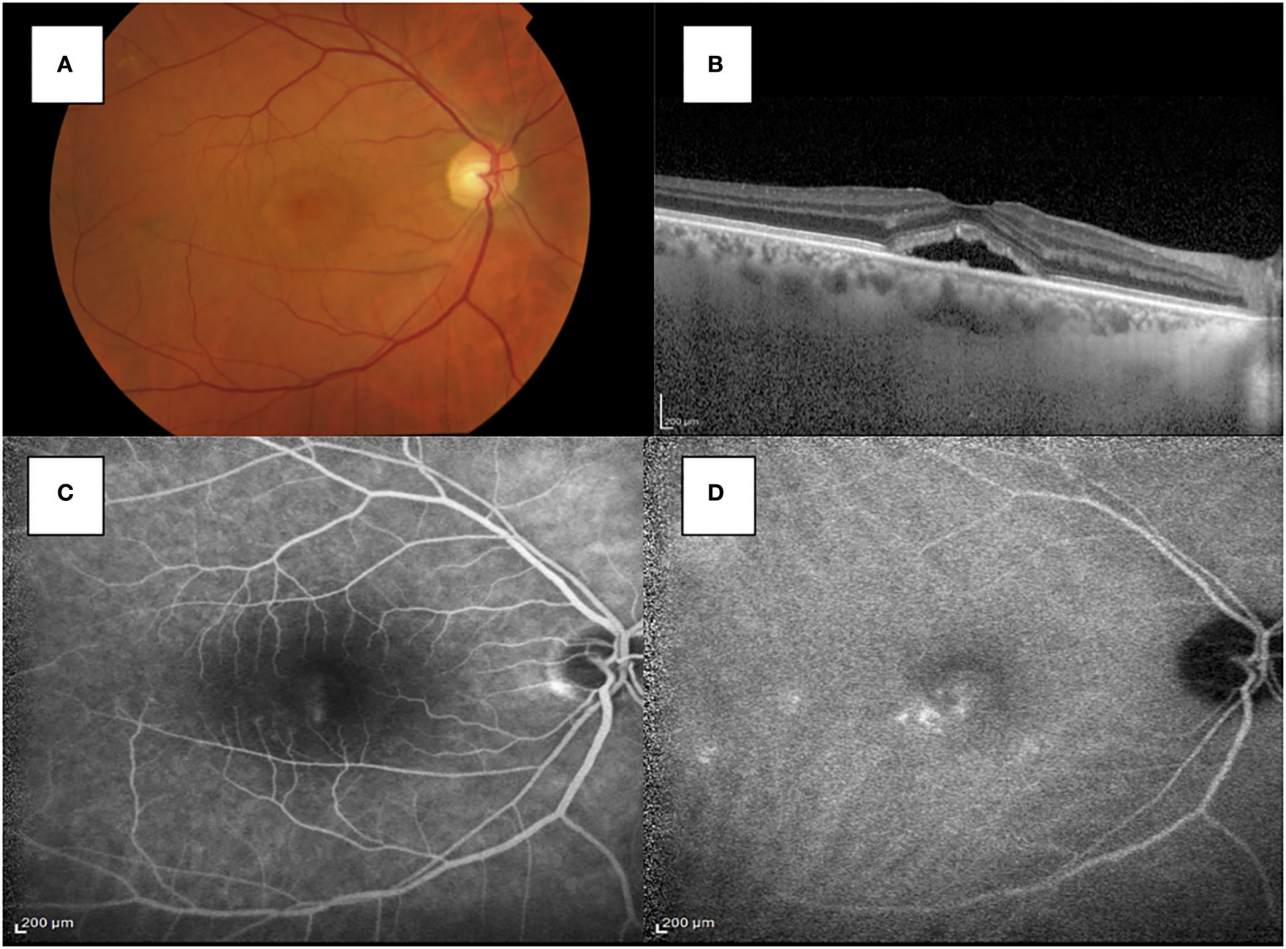
**(A)** Fundus photography showed the macular serous retinal detachment in the right eye. **(B)** SD-OCT shows the accumulation of subretinal fluid in the subfoveal region of the right eye. **(C)** The FA image showed spots of hyperfluorescence with a smokestack pattern in the late stage. **(D)** ICGA showed multiple spots of hypercianescence in the macular region.

After 2 months, the patient presented with a visual acuity of 20/30 in his right eye and an increase of subretinal fluid (SFR) in his right eye. Foveal thickness was 547 micrometers, whereas subfoveal choroidal thickness (SFCT) was 333 micrometers. Fluorescein angiography (FA) and indocyanine green angiography (ICGA) were performed and spots of macular leakage were observed (see Figure 2).

Considering all these findings, it was first hypothesized that the lesion in the right eye could be suggestive of an atypical case of chronic CSC, whose pathogenesis could be related to CADASIL.

The patient was not treated with oral medication and he was considered eligible for transfoveal subthreshold micropulse yellow laser (577 nm) therapy in his right eye (Iridex IQ577) (2).

Two weeks after laser treatment, SRF was completely reabsorbed with the presence of a flat irregular pigmented epithelial detachment, albeit without clinical implications; indeed, visual acuity was 20/25 in his right eye, with no complaining of visual symptoms. No adverse events were observed. At 1 month, visual acuity was 20/20, there were no signs of SRF, and both foveal thickness and SFCT were decreased to 247 micrometers and 316 micrometers, respectively.

**Figure 3:**
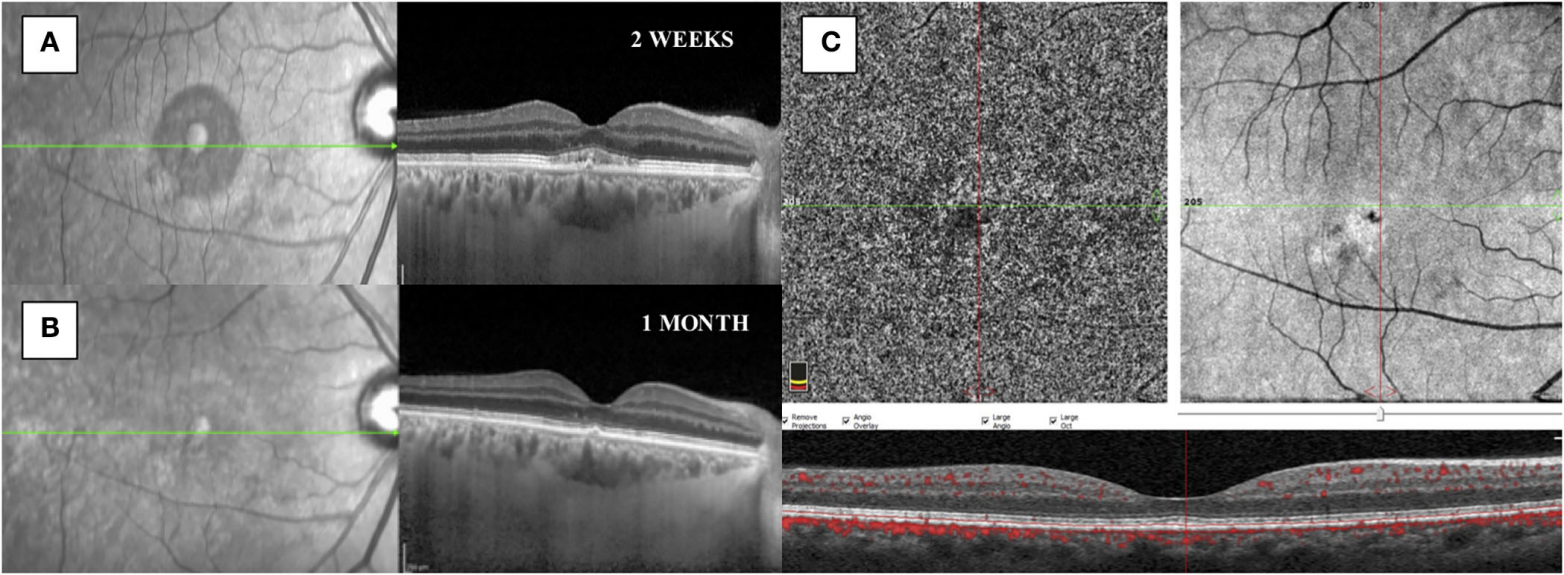
**(A)** SD-OCT performed 2 weeks after subthreshold micropulse yellow laser therapy showed a reduction of the subretinal fluid with the presence of a flat irregular pigmented epithelial detachment and granular subfoveal material. **(B)** SD-OCT showed a complete resolution of the subretinal fluid at 1 month. **(C)** OCT-A showed no signs of choroidal neovascularization after 1 month from treatment.

**Figure 4:**
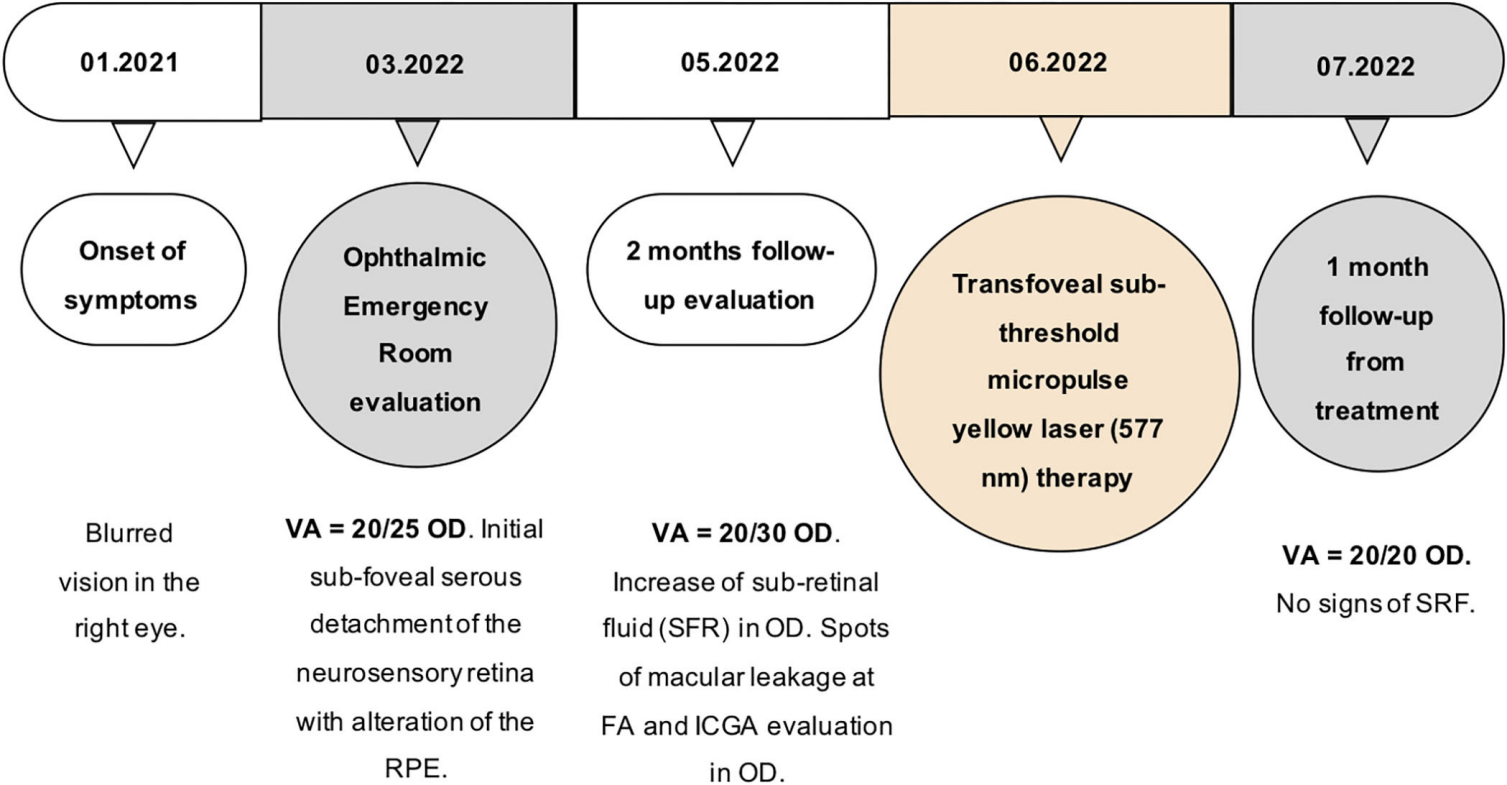
Timeline of the main events that occurred to the patient. OD, oculus dexter; nm, nanometer; SRF, sub-retinal fluid.

Optical coherence tomography angiography (OCT-A) (AngioVue Imaging System; Optovue, Inc., USA) was performed and no signs of neovascularization were observed in the choriocapillaris layer (see Figure 3). The brief timeline of our case is described in Figure 4.

## Discussion

Cerebral autosomal dominant arteriopathy with subcortical infarcts and leucoencephalopathy (CADASIL) is a rare inherited disease with almost exclusively neurological manifestations.

It usually affects middle-aged patients and its typical clinical manifestations include migraine, cognitive impairment, and recurrent strokes, leading to dementia and disability (3).

Ophthalmologically, patients with CADASIL may present visual aura with or without migraine, episodes of transient visual loss, and diplopia (4, 5).

Several studies demonstrated that CADASIL is also associated with retinal vascular abnormalities, resulting predominantly in the bilateral arteriolar sheathing, narrowing of arterioles, and arteriovenous nicking, secondary to accumulation of Notch3 domain in the vascular smooth cells and pericytes in the retinal vessels (6). A recent study by Lin et al. (7) using OCT-A showed that patients with CADASIL and a history of stroke had reduced macular vessel density in the superficial retinal plexus and reduced inner retinal thickness compared with the patients with non-stroke CADASIL. Also, they found a correlation between these two parameters and gait speed and the number of lacunae at MRI (7).

Moreover, Parisi et al. (8) observed a reduction of ERG, oscillatory potentials (OPs), pattern electroretinogram (PERG) amplitudes, and a reduction in VEP implicit time in patients with CADASIL, suggesting a dysfunction in the outer, middle, and innermost retinal layers.

In the present case, the patient was originally from Central Italy and he presented the mutation C.3016C>T in the exon 19 of gene Notch3, responsible for the protein change p.Arg1006Cys.

Bianchi et al. (9) conducted a retrospective study in a large group of Italian patients with CADASIL in Central Italy, where they observed that the most frequent mutation was p.Arg1006Cys, reported in 16.1% of patients. Also, they found that migraine with aura was the most frequent presenting symptom in their cohort of patients (59%), followed by TIA or stroke (51%) (9).

However, our patient did not present any neurological and ophthalmological signs or symptoms, except for the blurred vision in his right eye secondary to the development of CSC.

The involvement of choroidal vasculature in patients with CADASIL is still controversial.

Haritoglou et al. (10), based on a histopathological evaluation of two patients with CADASIL, showed an accumulation of granular material in retinal vessels, whereas choroidal vessels were not involved. Moreover, Alten et al. (11) showed no evidence of choroidal hypoperfusion and ischemia at OCT and ICGA in a cohort of 14 patients with CADASIL, compared with an age-matched control group. Instead, Robinson et al. (12) described irregular choroidal filling at the FA in patients with CADASIL suggesting a possible involvement of the choroidal vasculature.

Also, Rufa et al. (13) reported a case of nonarteritic anterior ischemic optic neuropathy (NAION) in a patient with CADASIL, secondary to a reduced blood flow in the anastomotic circle of the optic nerve head.

Furthermore, Fang et al. (14) showed a reduced subfoveal choroidal thickness in 27 patients with CADASIL at EDI-OCT, compared with an age-matched control group. Recently, Geerling et al. showed that patients with cerebral small vessels diseases have reduced choriocapillaris reflectivity compared with healthy controls at OCT-A, suggesting an alteration in the choroidal microvasculature (15).

Central serous chorioretinopathy (CSC) is characterized by localized serous detachment of the sensory retina, secondary to an alteration of the choriocapillaris and retinal pigmented epithelium (RPE). Central serous chorioretinopathy (CSC) belongs to the pachychoroid spectrum disorders, characterized by the presence of diffuse or localized choroidal thickening, increased diameter of choroidal vessels in Haller's layer, and thinning of inner choroidal vessels at Sattler's layer, and choriocapillaris.

Several studies suggested the role of choroidal ischemia in the pathogenesis of CSC, as reduced perfusion in the choriocapillaris seems to induce a functional alteration of the RPE, a hyperpermeability of choroidal vessels, and an increase of hydrostatic pressure in the interstitial space, leading to accumulation of fluid in the subretinal space (16).

Anatomically, the choroid is made of five layers: the Bruch membrane, the choriocapillaris, two vascular layers (such as Haller's and Sattler's), and the suprachoroidal. The main function of the choroid is to supply oxygen and nutrients to the outer retina. The vascular region of the choroid is made by the outer Haller's layer, consisting of large blood vessels, and the inner Sattler's layer with arterioles. Each arteriole gives rise to a lobular group of fenestrated capillaries in the choriocapillaris layer. The arterioles in Sattler's layer present vascular smooth muscle cells that are innervated by both divisions of the autonomic nervous system (17).

Histopathologically, CADASIL is characterized by the accumulation of the extracellular domain of Notch3 as granular osmiophilic material deposits in the cerebral and retinal arteriolar walls and precapillary arterioles, determining fibrosis and thickening of the vessels involved, eventually resulting in lumen occlusion.

We hypothesize that CADASIL could also be responsible for altering the vascular smooth cells in the choroidal arterioles, leading to subsequent chronic hypoperfusion and ischemia in the choriocapillaris. Although the association between CADASIL and CSC could also be accidental, the patient did not have any known risk factors for CSC and did not present any signs of pachychoroid and pachyvessels at the OCT examination that could explain the onset of the subretinal fluid.

Hence, we assume that choriocapillaris ischemia determined by CADASIL was our patient's main pathophysiological mechanism responsible for the onset of CSC.

To conclude, we believe that CADASIL should be considered a risk factor for the development of CSC, even without a pachychoroid spectrum.

## Conclusion

Cerebral autosomal dominant arteriopathy with subcortical infarcts and leucoencephalopathy (CADASIL) is a systemic disease that might affect choroidal vascular smooth cells, leading to chronic ischemia in the choriocapillaris and increasing the risk of developing CSC, even in the absence of a pachychoroid spectrum. Therefore, we believe that further studies should investigate the role of CADASIL in the alteration of choroidal vasculature to assess the risks of subsequent ophthalmological complications and treat these patients promptly.

## Data availability statement

The raw data supporting the conclusions of this article will be made available by the authors, without undue reservation.

## Ethics statement

Ethical review and approval was not required for the study on human participants in accordance with the local legislation and institutional requirements. The patients/participants provided their written informed consent to participate in this study. Written informed consent was obtained from the individual(s) for the publication of any potentially identifiable images or data included in this article.

## Author contributions

All authors agree that they have met the criteria for authorship, agree to the conclusions of this study, and agree to be accountable for the content of the work.

## Conflict of interest

The authors declare that the research was conducted in the absence of any commercial or financial relationships that could be construed as a potential conflict of interest.

## Publisher's note

All claims expressed in this article are solely those of the authors and do not necessarily represent those of their affiliated organizations, or those of the publisher, the editors and the reviewers. Any product that may be evaluated in this article, or claim that may be made by its manufacturer, is not guaranteed or endorsed by the publisher.
